# Supplementary material for: Content-rich biological network constructed by mining PubMed abstracts
Source: BMC Bioinformatics. 2004 Oct 8;5:147. doi: 10.1186/1471-2105-5-147 (PMC528731; doi:10.1186/1471-2105-5-147)
Supplement: Additional File 2 — The original results of the above study (non-essential files are deleted to keep the file size under the limit set by BMC bioinformatics). [file 1471-2105-5-147-S2.bz2 › chilibotAdditionalFile2/dip20/227ID7954478E744/html/CTNNB1_CTNNA1.html]

 


 **CTNNB1** and **CTNNA1** 
  
Found 3187 abstracts in PubMed, retrieved 20.  
 

 What does Google say? 
 PDF only 
| .edu only 

---

**Interactive relationship** (e.g. stimulation, inhibition, etc)

**Non-interactive relationship** (e.g. studied together, co-existance, homology, etc.)

- Expression of E cadherin, alpha catenin  [ **CTNNA1** ]   [ **CTNNB1** ] , and beta catenin  [ **CTNNA1** ]   [ **CTNNB1** ]  in the process of lymph node metastasis in oral squamous cell carcinoma.  Ref: 12888830 Br J Cancer, 2003
- TGF beta1 mediated MAP kinase activation also controls Wnt 7a gene expression and Wnt mediated signaling through the intracellular beta catenin  [ **CTNNA1** ]   [ **CTNNB1** ]  TCF pathway, which likely regulates N cadherin expression and subsequent N cadherin mediated cell adhesion complexes during the early steps of MPC chondrogenesis.  Ref: 12893825 J Biol ChemJ Biol Chem, 2003
- In support of this we show that separation of thymocytes from Wnt producing epithelial cells and the thymic microenvironment, triggers beta catenin  [ **CTNNA1** ]   [ **CTNNB1** ]  phosphorylation and degradation in thymocytes.  Ref: 12884861 Eur J Immunol, 2003
- Src activity was increased in QLalpha12 MDCK cells as assessed by Src autophosphorylation, and beta catenin  [ **CTNNA1** ]   [ **CTNNB1** ]  tyrosine phosphorylation was also increased, although there was no detectable increase in Rho activity.  Ref: 12890651 Am J Physiol Cell PhysiolAm J Physiol Cell Physiol, 2003
- the latter suppresses beta catenin  [ **CTNNA1** ]   [ **CTNNB1** ]  by promoting its degradation through enhanced inactivation of glycogen synthase kinase 3 GSK3.  Ref: 12883684 Oncol RepOncol Rep, 2003
- Thus, sustained exposure to Wnt in the context of an intact stromal microenvironment is necessary for stabilization of beta catenin  [ **CTNNA1** ]   [ **CTNNB1** ]  mediated signaling in thymocytes.  Ref: 12884861 Eur J Immunol, 2003
- Western blotting was used to determine polypeptide size and expression of E cadherin and beta catenin  [ **CTNNA1** ]   [ **CTNNB1** ]  in Wilms s tumours compared with normal kidney.  Ref: 12890743 Mol Pathol, 2003
- Identification of genes differentially expressed in mouse mammary epithelium transformed by an activated beta catenin  [ **CTNNA1** ]   [ **CTNNB1** ] .  Ref: 12881717 Oncogene, 2003
- Low to high gamma catenin  [ **CTNNA1** ]   [ **CTNNB1** ]  and ezrin expression was seen in cytoplasm of blastemal and epithelial cells of primary and metastatic tumours.  Ref: 12890743 Mol Pathol, 2003
- beta Catenin  [ **CTNNA1** ]   [ **CTNNB1** ]  is an executor of Wnt signaling and it can control cell fate and specification.  Ref: 12881717 Oncogene, 2003
- OBJECTIVE To study the relationship between the abnormal expression of beta catenin  [ **CTNNA1** ]   [ **CTNNB1** ]  beta cat and the high expressions of cyclin D1 and c myc and the occurance, proliferation, infiltration, metastasis and prognosis of pancreatic cancer, and to provide rational basis for the clinical diagosis and treatment.  Ref: 12882690 Zhonghua Bing Li Xue Za Zhi, 2003
- beta catenin  [ **CTNNA1** ]   [ **CTNNB1** ]  is one of the essential components of this specification.  Ref: 12885771 J Biol ChemJ Biol Chem, 2003
- The distribution of plakoglobin and beta catenin  [ **CTNNA1** ]   [ **CTNNB1** ]  was determined by immunofluorescence, western blot analysis, pulse chase radiolabeling, and biochemical subcellular fractionation.  Ref: 12880414 J Invest Dermatol, 2003
- Cellular retinol binding protein 1 in hepatocellular carcinoma correlates with beta catenin  [ **CTNNA1** ]   [ **CTNNB1** ] , Ki 67 index, and patient survival.  Ref: 12883492 HepatologyHepatology, 2003
- Distribution of cell cell adhesion molecules of E cadherin EC and beta catenin  [ **CTNNA1** ]   [ **CTNNB1** ]  BC infiltrating breast carcinoma BC and markers of epithelium, basal membrane has been studied by immunofluorescence in 50 infiltrating BC tissue samples including 17 ductal carcinomas, 23 lobular carcinomas and 10 combined carcinomas.  Ref: 12879601 Arkh PatolArkh Patol, 2003
- p120 catenin  [ **CTNNA1** ]   [ **CTNNB1** ]  p120 was originally identified as a tyrosine kinase substrate, and subsequently shown to regulate cadherin mediated cell cell adhesion.  Ref: 12885254 Biochemistry, 2003
- Here we show that beta catenin  [ **CTNNA1** ]   [ **CTNNB1** ]  signaling is required for the formation of the distal, but not the proximal, airways.  Ref: 12885771 J Biol ChemJ Biol Chem, 2003
- CONCLUSIONS Compared with primary tumours, metastatic tumours showed lower expression of E cadherin and gamma catenin  [ **CTNNA1** ]   [ **CTNNB1** ] , with nuclear staining for beta catenin  [ **CTNNA1** ]   [ **CTNNB1** ] .  Ref: 12890743 Mol Pathol, 2003
- These results are consistent with a signaling pathway initiated by structural changes in the adherens junction in which adherens junction derived plakoglobin regulates nuclear transcription by antagonizing the binding of beta catenin  [ **CTNNA1** ]   [ **CTNNB1** ]  to TCF LEF proteins.  Ref: 12880414 J Invest Dermatol, 2003
- We have studied the effects of the organophosphorous pesticide malathion on cell viability, actin cytoskeleton, cell adhesion complex E cadherin beta catenin  [ **CTNNA1** ]   [ **CTNNB1** ] , and Rho and Rac1 GTPases from the human mammary carcinoma cell line MCF 7.  Ref: 12888906 Int J Oncol, 2003
- Primary and metastatic tumours showed moderate to high beta catenin  [ **CTNNA1** ]   [ **CTNNB1** ]  expression in blastemal and epithelial cells, with predominantly membranous and cytoplasmic staining.  Ref: 12890743 Mol Pathol, 2003
- To investigate the existence of adherens junction mediated signaling, we used peroxovanadate to tyrosine phosphorylate plakoglobin and beta catenin  [ **CTNNA1** ]   [ **CTNNB1** ]  and to dissociate adherens junctions.  Ref: 12880414 J Invest Dermatol, 2003
- METHODS Immunohistochemistry was used to determine the expression and cellular distribution of E cadherin, beta catenin  [ **CTNNA1** ]   [ **CTNNB1** ] , gamma catenin  [ **CTNNA1** ]   [ **CTNNB1** ] , and ezrin in primary and metastatic Wilms s tumours.  Ref: 12890743 Mol Pathol, 2003
- These data suggest that evaluation of the immunohistochemical expression of E cadherin, alpha catenin  [ **CTNNA1** ]   [ **CTNNB1** ]  and beta catenin  [ **CTNNA1** ]   [ **CTNNB1** ]  is extremely valuable for the diagnosis of metastatic occurrence.British Journal of Cancer 2003 8 57 563.  Ref: 12888830 Br J Cancer, 2003
- Higher amounts of 92 kDa beta catenin  [ **CTNNA1** ]   [ **CTNNB1** ]  were detected in tumours than in normal kidney.  Ref: 12890743 Mol Pathol, 2003
- Nuclear translocation was associated with increased plakoglobin and decreased beta catenin  [ **CTNNA1** ]   [ **CTNNB1** ]  binding to nuclear TCF LEF and downregulation of gene transcription from TCF LEF reporter constructs.  Ref: 12880414 J Invest Dermatol, 2003
- Mutation analysis of  **CTNNB1**  beta catenin  [ **CTNNA1** ]   [ **CTNNB1** ]  and AXIN1, the components of Wnt pathway, in cervical carcinomas.  Ref: 12883680 Oncol RepOncol Rep, 2003
- In the metastatic process, reduction in cell to cell adhesion including E cadherin catenin  [ **CTNNA1** ]   [ **CTNNB1** ]  cell adhesion complex is an essential step.  Ref: 12888830 Br J Cancer, 2003
- Deletion of exon 3 from the endogenous beta catenin  [ **CTNNA1** ]   [ **CTNNB1** ]  gene in differentiating mammary alveolar epithelium of the mouse results in the generation of an activated protein that lacks amino acids 5 80.  Ref: 12881717 Oncogene, 2003
- Because changes in cell cell adhesion have profound effects on cellular behavior, we hypothesized a link between the adhesion and signaling functions of plakoglobin and beta catenin  [ **CTNNA1** ]   [ **CTNNB1** ] .  Ref: 12880414 J Invest Dermatol, 2003
- To further understand the molecular process of transdifferentiation, the expression of genes in mammary tissue was profiled in the absence and presence of activated of beta catenin  [ **CTNNA1** ]   [ **CTNNB1** ] .  Ref: 12881717 Oncogene, 2003
- Significantly greater reduction in expression levels of E cadherin, alpha catenin  [ **CTNNA1** ]   [ **CTNNB1** ]  and beta catenin  [ **CTNNA1** ]   [ **CTNNB1** ]  was found in the metastatic group n=64 compared to the nonmetastatic group n=95 P=0.00.  Ref: 12888830 Br J Cancer, 2003
- Expression of non destructible beta catenin  [ **CTNNA1** ]   [ **CTNNB1** ]  mutants increased survivin expression and protected against ultraviolet B induced apoptosis.Interpretation Stimulation of survivin expression by TCF beta catenin  [ **CTNNA1** ]   [ **CTNNB1** ]  might impose a stem cell like phenotype to colonic crypt epithelium coupling enhanced cell proliferation with resistance to apoptosis, and contribute to the molecular pathogenesis of colorectal cancer.  Ref: 12885482 Lancet, 2003
- Coimmunoprecipitation studies from nuclear fractions, gel shift assays, and transient transfections with T cell factor TCF lymphoid enhancer factor LEF optimized promoter reporter constructs were used to investigate the ability of plakoglobin and beta catenin  [ **CTNNA1** ]   [ **CTNNB1** ]  that had redistributed from the membrane to the nucleus to form functional transcriptional regulatory complexes with TCF LEF family member transcription factors.  Ref: 12880414 J Invest Dermatol, 2003
- Prognosis did not correlate with the nuclear beta catenin  [ **CTNNA1** ]   [ **CTNNB1** ]  expression.  Ref: 12883492 HepatologyHepatology, 2003
- beta catenin  [ **CTNNA1** ]   [ **CTNNB1** ]  is required for specification of proximal distal cell fate during lung morphogenesis.  Ref: 12885771 J Biol ChemJ Biol Chem, 2003
- Tyrosine phosphorylation of plakoglobin and beta catenin  [ **CTNNA1** ]   [ **CTNNB1** ]  resulted in their rapid translocation from the cell membrane to the nucleus.  Ref: 12880414 J Invest Dermatol, 2003
- This is supported by the finding that beta catenin  [ **CTNNA1** ]   [ **CTNNB1** ]  known to be overexpressed in JAs acts as a co activator of the AR.  Ref: 12883689 Oncol RepOncol Rep, 2003
- Accumulating evidence has highlighted the importance of beta catenin  [ **CTNNA1** ]   [ **CTNNB1** ]  mediated activation of T cell factor TCF lymphoid enhancer factor LEF transcription during thymocyte development.  Ref: 12884861 Eur J Immunol, 2003
- Changes in apoptosis were monitored in cell lines engineered to express stabilising mutations in beta catenin  [ **CTNNA1** ]   [ **CTNNB1** ] .Findings TCF beta catenin  [ **CTNNA1** ]   [ **CTNNB1** ]  stimulated a six fold to 12 fold increased expression of the survivin gene in colorectal cancer cells.  Ref: 12885482 Lancet, 2003
- In this chapter, we will review the current status of dynamic glycosylation phosphorylation of several important regulatory proteins including c Myc, estrogen receptors, Sp1, endothelial nitric oxide synthase, and beta catenin  [ **CTNNA1** ]   [ **CTNNB1** ] .  Ref: 12882516 Prog Nucleic Acid Res Mol Biol, 2003
- We investigated immunohistochemical expression of E cadherin, alpha catenin  [ **CTNNA1** ]   [ **CTNNB1** ]  and beta catenin  [ **CTNNA1** ]   [ **CTNNB1** ]  in 159 tissue samples from patients with oral squamous cell carcinoma and examined the correlation between their expressions and the presence of regional lymph node metastasis.  Ref: 12888830 Br J Cancer, 2003
- When the gene for beta catenin  [ **CTNNA1** ]   [ **CTNNB1** ]  was conditionally excised in epithelial cells of the developing mouse lung prior to E14.5, the proximal lung tubules grew and differentiated appropriately.  Ref: 12885771 J Biol ChemJ Biol Chem, 2003
- AIMS To evaluate E cadherin, beta catenin  [ **CTNNA1** ]   [ **CTNNB1** ] , gamma catenin  [ **CTNNA1** ]   [ **CTNNB1** ] , and ezrin expression and its relation to histopathological features of primary and metastatic Wilms s tumours.  Ref: 12890743 Mol Pathol, 2003
- Immunocharacterization with beta catenin  [ **CTNNA1** ]   [ **CTNNB1** ] , E cadherin, cytokeratins AE1 AE3, epithelial membrane antigen, S 100 protein, smooth muscle actin, and vimentin was also performed.  Ref: 12883237 Am J Surg Pathol, 2003
- BACKGROUND The E cadherin catenin  [ **CTNNA1** ]   [ **CTNNB1** ]  adhesion complex is crucial for intercellular adhesiveness and maintenance of tissue architecture.  Ref: 12890743 Mol Pathol, 2003
- beta Catenin  [ **CTNNA1** ]   [ **CTNNB1** ]  mutations in liver tumors induced by 2 amino dimethylimidazo f quinoline in CDF 1 mice.  Ref: 12893427 Cancer Lett, 2003
- Adhesion Associated and PKC Modulated Changes in Serine Threonine Phosphorylation of p120 Catenin  [ **CTNNA1** ]   [ **CTNNB1** ] .  Ref: 12885254 Biochemistry, 2003
- These changes were related to a decrease in the expression of the adhesion molecules, E cadherin and beta catenin  [ **CTNNA1** ]   [ **CTNNB1** ] , and to the distribution and reactivity of actin microfilaments to TRITC phalloidin.  Ref: 12888906 Int J Oncol, 2003
- Background Colorectal cancer is thought to originate in the expansion of colonic crypt cells as a result of aberrant gene expression caused by transcription factors of the T cell factor TCF beta catenin  [ **CTNNA1** ]   [ **CTNNB1** ]  family.  Ref: 12885482 Lancet, 2003
- In this study, we screened liver tumors induced by 2 amino dimethylimidazo f quinoline MeIQ in CDF 1 mice for beta catenin  [ **CTNNA1** ]   [ **CTNNB1** ]  and APC mutations and other genetic alterations shown to occur in human hepatocellular carcinomas HCC, including mutations in the p53 and H ras genes, c myc amplification and E cadherin promoter methylation.  Ref: 12893427 Cancer Lett, 2003
- There was a close correlation between nuclear CRBP 1 inclusions and nuclear beta catenin  [ **CTNNA1** ]   [ **CTNNB1** ]  staining in HCCs P =.008, suggesting a cross talk between CRBP 1 and the Wnt wingless signal transduction pathway.  Ref: 12883492 HepatologyHepatology, 2003
- These data indicate that except for the occasional disruption of the Wnt pathway through beta catenin  [ **CTNNA1** ]   [ **CTNNB1** ]  mutations, the genetic pathways involved in the development of HCC differ significantly between human liver cancer and tumors induced in mice by MeIQ, but do not rule out the possibility that heterocyclic amines constitute a carcinogenic risk factor in humans.  Ref: 12893427 Cancer Lett, 2003
- SSCP followed by direct DNA sequencing revealed mutations in exon 2 of the beta catenin  [ **CTNNA1** ]   [ **CTNNB1** ]  gene in 2 of 16 liver tumors 12.5%.  Ref: 12893427 Cancer Lett, 2003
- Differential expression of E cadherin and beta catenin  [ **CTNNA1** ]   [ **CTNNB1** ]  in primary and metastatic Wilms s tumours.  Ref: 12890743 Mol Pathol, 2003
- Lastly, stabilization of beta catenin  [ **CTNNA1** ]   [ **CTNNB1** ]  resulted in the retention of differentiated epithelium upon involution and altered activities of several proteases in transdifferentiated mammary epithelium.Oncogene 2003 2 594 4610.  Ref: 12881717 Oncogene, 2003
